# Heat-Stable Molecule Derived from *Streptococcus cristatus* Induces APOBEC3 Expression and Inhibits HIV-1 Replication

**DOI:** 10.1371/journal.pone.0106078

**Published:** 2014-08-28

**Authors:** Ziqing Wang, Yi Luo, Qiujia Shao, Ballington L. Kinlock, Chenliang Wang, James E. K. Hildreth, Hua Xie, Bindong Liu

**Affiliations:** 1 Center for AIDS Health Disparities Research, School of Medicine, Meharry Medical College, Nashville, Tennessee, United States of America; 2 Department of Oral Biology and Research, School of Dentistry, Meharry Medical College, Nashville, Tennessee, United States of America; 3 Institute of Gastroenterology and Institute of Human Virology, Sun Yat-sen University, Guangzhou, Guangdong, Peoples of Republic of China; University Hospital Zurich, Switzerland

## Abstract

Although most human immunodeficiency virus type 1 (HIV-1) cases worldwide are transmitted through mucosal surfaces, transmission through the oral mucosal surface is a rare event. More than 700 bacterial species have been detected in the oral cavity. Despite great efforts to discover oral inhibitors of HIV, little information is available concerning the anti-HIV activity of oral bacterial components. Here we show that a molecule from an oral commensal bacterium, *Streptococcus cristatus* CC5A can induce expression of APOBEC3G (A3G) and APOBEC3F (A3F) and inhibit HIV-1 replication in THP-1 cells. We show by qRT-PCR that expression levels of A3G and A3F increase in a dose-dependent manner in the presence of a CC5A extract, as does A3G protein levels by Western blot assay. In addition, when the human monocytic cell line THP-1 was treated with CC5A extract, the replication of HIV-1 IIIB was significantly suppressed compared with IIIB replication in untreated THP-1 cells. Knock down of A3G expression in THP-1 cells compromised the ability of CC5A to inhibit HIV-1 IIIB infectivity. Furthermore, SupT1 cells infected with virus produced from CC5A extract-treated THP-1 cells replicated virus with a higher G to A hypermutation rate (a known consequence of A3G activity) than virus used from untreated THP-1 cells. This suggests that *S. cristatus* CC5A contains a molecule that induces A3G/F expression and thereby inhibits HIV replication. These findings might lead to the discovery of a novel anti-HIV/AIDS therapeutic.

## Introduction

HIV-1 can be transmitted through intravenous contact with contaminated blood products, from infected mothers to offspring *in utero* or through lactation after birth. However, the predominant mode of HIV transmission is by sexual contact through broken mucosal or epidermal epithelia. In contrast, oral transmission of HIV is an extremely rare event [1]–[3]. This is true despite the fact that a recent study reported that cell-associated proviral HIV-1 DNA was detected in gingival crevicular fluid of 49% of HIV-1 infected patients [4]. Moreover, hyper-excretion of HIV-1 RNA in saliva has been reported [5]. Normally HIV-1 infected patients have plasma titers exceeding those found in saliva, but in one study, 7% of 67 tested individuals had a four-fold or higher viral load in saliva compared to plasma [5]. Therefore, it has been suggested that certain oral components are capable of inactivating HIV-1. Several endogenous components have been proposed as contributors to HIV inactivation, either when found alone or in combination [6]. Among them, secretory leucocyte protease inhibitor (SLPI) has been extensively studied [7]. Another known class of proteins with innate immunity capacity in the oral cavity is the β-defensins, which are secreted by epithelial cells [8].

More than 700 bacterial species have been detected in the oral cavity [9]. Despite great effort to uncover oral inhibitors of HIV, little information is available on the anti-HIV activity of oral bacterial components. Our previous work revealed that the binding domains (HGP17 and 44) of *Porphyromonas gingivalis’* gingipains can block HIV infection by interacting with the viral glycoprotein, gp120 [10]. One mechanism by which products from oral bacteria could block HIV infection that has not been considered is their role in the production of host cell restriction factors. One such factor is APOBEC3. Here we report that *S. cristatus* expresses a small molecular weight heat-stable molecule with apparent anti-HIV activity via its ability to induce expression of the human HIV-restriction factor APOBEC. This ability is previously unreported in bacteria.

*S. cristatus* is a member of the mitis group of streptococci and has been detected on the surfaces of the buccal epithelium and the teeth of healthy individuals [9]. It has not been associated with oral infectious diseases, although it has been documented that noninvasive *S. cristatus* can be transferred into human epithelial cells by *F. nucleatum* [11]. Interestingly, a small study of 14 HIV-1 positive patients by the Forsyth Institute examined the presence/absence of 109 oral bacterial species. The patients suffered from gingivitis, periodontitus or linear gingival erythema. The authors found detectable levels of *S. cristatus* in 2 of 9 HIV-positive patients who had a low viral load and high CD4 counts, while *S. cristatus* was undetectable in all of the 5 patients with high viral load and low CD4 counts [12]. However at this time it is premature to make any conclusions about the correlation between specific bacterial species and the different immune states of HIV-positive patients.

Members of the APOBEC3 family of proteins that mediate cytidine deaminase activity, serve as potent restriction factors to HIV-1 infection [13]–[15]. Among them, APOBEC3G (A3G) and ABOPEC3F (A3F) show potent anti-HIV-1 activity [15]–[20]. HIV-1 Vif is a 23 kD small regulatory protein which is essential for HIV-1 replication [21], [22]. HIV-1 Vif hijacks human ubiquitin E3 ligase Cullin 5 [23] to promote the proteosome mediated degradation of A3G [23]–[28]. Degradation of A3G depletes A3G from the cytoplasm of virus-producing cells thereby preventing A3G from being incorporated into virions to perform its antiviral function. In the absence of Vif, A3G is packaged into the HIV-1 virion [23], [25]–[29]. Upon infecting the target cells, A3G drastically restricts HIV-1 replication by causing G to A hypermutations in the newly synthesized cDNA. Recent reports show that A3G also inhibits the production of HIV-1 reverse transcription products [30]–[33] and viral DNA integration [32] and that antiviral activity found in A3G could also be independent of its deaminase activity [33], [34]. In addition to inhibition of HIV-1, A3G restricts replication of other retroviruses including simian immunodeficiency virus, equine infectious anemia virus and murine leukemia virus. Similar in activity to A3G, [17], [19], [20], [35] A3F is also packaged into HIV-1ΔVif virions and induces G to A hypermutations, although it differs with respect to the target sequence motif [19].

A3G gene expression is upregulated in response to multiple regulatory factors. Treatment of H9 T cell lines with phorbol myristate acetate (PMA) increases expression of A3G. This increased expression of A3G is mediated by the protein kinase C/mitogen-activated protein/ERK signaling cascade [36]. Similarly, treatment of resting peripheral blood lymphocytes (PBLs) with phytohemagglutinin (PHA) plus IL-2 or particular cytokines when each are added alone (IL-2, IL-7 and IL-15) increases expression of A3G through the JAK/MAPK signaling pathway [24], [37]. Interferon also plays a role in upregulating A3G in primary macrophages, CD4 T cells and primary hepatocytes or in laboratory-adapted hepatocellular carcinoma cell lines such as Hep3B, HepG2 and Huh 7 [38]–[40]. Here we will show evidence that a molecule associated with *S. cristatus* can induce expression of A3G and A3F and inhibit HIV-1 replication.

## Materials and Methods

### Cell culture and reagents

The cell lines THP-1, SupT1, TZM-bl and H9/HTLV- IIIB along with anti-human APOBEC3G and anti-human APOBEC3F antibody were obtained from the NIH AIDS Research and Reference Reagent Program. THP-1 and Sup T1 were cultured in regular RPMI medium with 10% fetal calf serum (FBS). TZM-bl was grown in DMEM supplemented with 10% fetal calf serum. Immortalized human keratinocytes OKF6/TERT-2, provided by Dr. James G. Rheinwald (Harvard Medical School, MA) [41], were cultured in Keratinocyte-SFM (Invitrogen) supplemented with 0.2 ng/ml recombinant epidermal growth factor (rEGF; Invitrogen), 25 µg/ml bovine pituitary extract (BPE) and 0.4 mM CaCl2 (final). Immortalized human vaginal epithelial cells VK2/E6E7, purchased from ATCC and cultured as described [42]. Lipopolysaccharide (LPS-EK Ultrapure) and lipoteichoic acid (LTA-SA) were purchased from Invivogen. MEK inhibitor U0126 was purchased from Calbiochem. Anti p-ERK (E-4) (sc-7383) and anti-ERK2 (D-2) (sc-1647) were purchased from Santa Cruz, USA.

### Preparation of sonicated bacterial cell extracts

Oral bacteria, including *Actinomyces naeslundii* NC-3, *Streptococcus gordonii* DL1, *S. cristatus* CC5A, *S. mutans* KPSK2, *Actinomyces viscosus* EG4, and *Porphyromonas gingivalis* 33277, which were from our laboratory collection at Meharry Medical College School of Dentistry, were grown in appropriate media aerobically or anaerobically at 37°C for 16 h. Bacterial cells were harvested by centrifugation at 6,000×*g* for 10 min at 4°C. Bacterial cells were washed twice with cold PBS. The cell extracts were then prepared by sonication at Power 15 (10×30 sec) using a Misonix XL-2000. The supernatants were collected and filtered through a 0.22 µm PVDF filter (Millipore). The bacterial extracts were stored in −20°C freezers if long term storage were needed.

### Preparation of the *S. cristatus* PBS solution

*S. cristatus* CC5A cells were incubated in PBS at 37°C for 4 h without sonication, and the supernatant of the cell mixture (hereafter termed CC5A/PBS) was collected following centrifugation at 6,000*g,* 4°C, for 15 min and filtration through a 0.22 µm PVDF filter (Millipore). For separate experiments, some *S. cristatus* CC5A cells were also boiled in 2% SDS for 1 h. After washing them six times with PBS, the cells were either incubated in PBS at 37°C for 4 h for preparation of a CC5A/PBS solution or they were sonicated as described to prepare a sonicated bacterial cell extracts.

To treat THP-1 cells, 25 µg (or as indicated) equivalent protein content measured by BCA assay (Pierce) of bacterial extracts was added to 5×10^5^ cells in 12-well-plates (Celltreat Scientific Products) containing 1 ml of complete medium and incubated at 37°C, 5% CO_2_ for 16 h. After treatment, THP-1 cells were subjected to total RNA isolation as indicated in the qRT-PCR procedure. For the MEK inhibitor treatment, 5×10^5^ THP-1 cells were treated with 20 µM U0126 for 1 h prior to CC5A/PBS treatment. After U0126 treatment, THP-1 cells were washed twice with PBS and subjected to CC5A/PBS treatment.

To treat OKF6 or VK2 cells, 100% confluent cells in a 12-well-plate (around 5×10^5^ cells per well) were treated with the indicated amount of CC5A/PBS for the indicated times. After treatment, cells were analyzed by either qRT-PCR or Western-blot as described previously [43].

### qRT-PCR

Real-time quantitative reverse transcription PCR (qRT-PCR) was performed with a Bio-Rad MyiQ Single-Color Real-Time PCR Detection System using Bio-Rad iQ SYBR Green Supermix reagent. Total RNA was extracted using a TRizol plus PureLink Micro-to-Midi Total RNA Purification System (Invitrogen) following the manufacturer’s protocol. DNA was further removed by on-column digestion using RQI RNase free DNase (Promega). An equal amount of RNA was reverse transcribed using random hexamer primers and M-MLV RT reverse transcriptase (Promega). Expression of A3F, A3G and GAPDH were analyzed by qRT-PCR using the following primers A3G: 5′-TCAGAGGACGGCATGAGACTTA-3′, 5′-AGCAGGACCCAGGTGTCATT-3′; A3F: 5′-CCTACGCAAAGCCTATGGTCGG-3′, 5′-CCAGGAGACAGGTGAGTGGTGC-3′; GAPDH: 5′-GAAGGTGAAGGTCGGAGT-3′, 5′-GAAGATGGTGATGGGATTTC-3′ as described previously [44]. qRT-PCR result was normalized using GAPDH amplification levels and calculated by 2^−ΔΔCT^ comparative method. All experiments were independently repeated at least three times. The variation was expressed by calculating the standard deviation (SD) from the three independent experiments and presented as the mean values ± SD.

### Gel filtration chromatography

D-Salt Polyacrylamide Desalting Columns (6KMWCO, Pierce) were used to estimate the molecular weight of the active molecule of *S. cristatus*. The column was mounted on a LKB FPLC system. The column was calibrated by a gel-filtration standard (Bio-rad Cat #151-1901); 100 µg CC5A/PBS was loaded onto the column. The chromatography was performed under the following conditions: elution buffer: PBS; flow rate: 1 ml/min; and UV monitoring: 220 nm. Fractions (1.5 ml) were collected and the induction effect of these fractions on A3G mRNA was measured by qRT-PCR.

### Viral infectivity assay

HIV-1 IIIB virus was obtained from the cell cultural supernatant of H9/HTLV-IIIB cell line. THP-1 cells were infected by HIV-1 IIIB virus and cultured for two weeks to generate a chronically infected THP-1 cell line (THP-1/IIIB). Equal numbers of these cells were treated with PBS or 40 µg CC5A/PBS overnight. After washing cells twice with PBS, cells were cultured in complete medium at 37°C, 5% CO_2_ for 24 h. After 24 h of culture, the cells were washed twice again with PBS, then cultured in the fresh complete medium for another 24 h. The culture supernatants were harvested. The released virus was measured by standard p24 ELISA assay. The viral infectivity of released virus was measured by a MAGI assay using TZM-bl cells as described [45]. Briefly, TZM-bl cells were infected with serial dilutions of released virus in complete medium with 20 µg/ml of DEAE-Dextran in a 37°C, 5% CO_2_ incubator. After 3 h incubation, the culture volume was increased 1 fold by complete medium. After 48 h incubation, viral infectivity was measured by a Promega Luciferase Kit. The luciferase result was normalized to the p24 input for each sample.

### Viral replication assay

THP-1 cells (5×10^5^) were treated with only PBS or 40 µg CC5A/PBS overnight. After washing twice with PBS, the treated cells were infected with equal amounts of HIV-1 IIIB (containing 100 ng p24 protein content) for 3 h. The cells were washed twice with PBS and cultured in complete medium for 8 days at 37°C, 5% CO_2_. Every other day, a 50 µl sample of supernatant was collected and p24 content determined using a standard p24 ELISA assay. A viral replication curve was generated by plotting p24 concentration against replication days.

### Establishment of A3G knockdown THP-1 cells using CRISPR/Case9 system

Oligos: CACCGCGAAGCGCCTCCTGGTAATC and AAACGATTACCAGGAGGCGCTTCGC were annealed together to form short dsDNA 1; oligos: CACCGTAACCTTCGGGTCCTCGGCC and AAACGGCCGAGGACCCGAAGGTTAC were annealed together to form short dsDNA 2. dsDNA 1 and dsDNA 2 were designed to target A3G genome exon 3. pX330 (Addgene 42230) was digested by BssI. dsDNA1 and dsDNA2 were cloned into pX330 respectively to form pX330-A3G1 and pX330-A3G2. pX330-A3G1 and pX330-A3G2 (3 µg each) were transfected into 1×10^6^ THP-1 cells using Neon Transfection System (Invitrogen). The transfection condition is voltage: 1300 V; width: 30 ms; pulse: 1 pulse. Three days post transfection, the transfected THP-1 cells were cloned by limited dilution method. Single clones were screen by Western-blot and qRT-PCR for A3G expression analysis.

### DNA hypermutation assay

Equal numbers of THP-1/IIIB cells were treated with PBS or 40 µg CC5A/PBS overnight. After washing with PBS twice, the cells were cultured in complete medium at 37°C, 5% CO_2_ for two days. Culture supernatants containing released virus (containing 100 ng p24 protein content) were used to infect 1×10^6^ Sup T1 cells for 4 h. The infected SupT1 cells were washed twice with PBS then incubated at 37°C, 5% CO_2_ for 16 h. DNA was isolated using DNeasy DNA isolation kit (Qiagen). A 650 bp DNA fragment covering a portion of nef, U3 and R of HIV-1 was amplified with platinum Taq DNA polymerase (Invitrogen) using the primers HIV-1-F, 5′-AGGCAGCTGTAGATATTAGCCAC, and HIV-1-R, 5′-GTATGAGGGATCTCTAGCTACCA. The PCR products were cloned into the TOPO TA-cloning vector (Invitrogen). The nucleotide sequences of individual clones from each infected culture sample were determined [46]. Statistical significance was determined by Mann Whitney U test using GraphPad Prism software.

### Cytotoxicity assay

The cytotoxicity of CC5A/PBS and Sonicated CC5A treatment was measured by a Live/Dead Cell Vitality Assay Kit (Invitrogen). The kit provides a two-color fluorescence assay that distinguishes metabolically active cells from injured cells and dead cells. THP-1 cells were treated with the indicated dose of protein from sonicated CC5A or unsonicated CC5A/PBS respectively. PBS treated THP-1 was used as untreated control. Sixteen h post treatment, the samples were subjected to Live/Dead Cell Vitality Assay Kit following the product manual. The samples were analyzed by BD FACSCalibur.

## Results

### Bacterial extract from *S. cristatus* CC5A induces A3F and A3G expression

**Figure 1:**
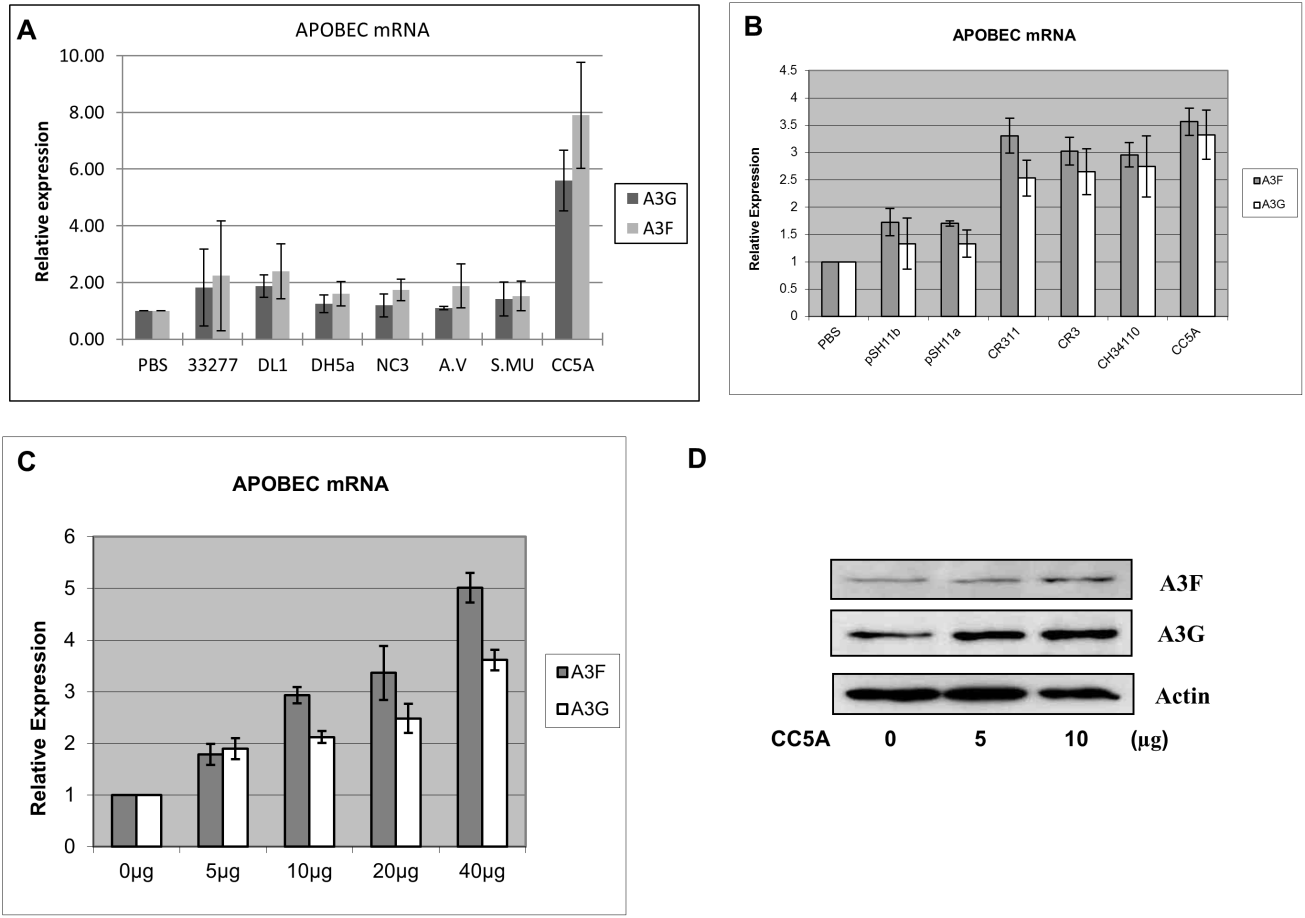
A molecule derived from oral *Streptococcus cristatus* induces APOBEC3F/G expression. (A) Cell extracts from NC3 (*Actinomyces naeslundii* NC-3), DL1 (*Streptococcus gordonii* DL1) and DH5a, CC5A (*S. cristatus* CC5A), A.V (*Actinomyces viscosus*), 33277 (*Porphyromonas gingivalis* 33277) and S.MU (*S. mutans* KPSK2) were used to treat THP-1 cells. THP-1 cells treated by PBS were used as a control. After 16 h of exposure, total RNA was isolated and the expression of A3G and A3F quantified by qRT-PCR. (B) CC5A and different strains of *S. cristatus* (pSH11b, pSH11a, CR311, CR3, and CH34110) were used to treat THP-1. The induction of A3G and A3F was measured by qRT-PCR. (C) Different doses (0–40 µg) of CC5A/PBS were used to treat THP-1 cells for 16 h. A3G and A3F expression was measured by qRT-PCR. (D) A3G and A3F expression following a 48 h exposure to 0–10 µg CC5A was tested by Western blot assay. Values shown in (A), (B) and (C) are given as means ± SD of three independent experiments compared to normalized to 1.0 for controls. The Western-blot data is representative of three independent experiments.

To determine the role of oral bacteria on the expression of innate intracellular immune factors A3G and A3F, we utilized a group of oral bacteria, including *A. naeslundii* NC-3, *S. gordonii* DL1, *S. cristatus* CC5A, *S. mutans* KPSK2, *A. viscosus,* and *P. gingivalis* 33277. The sonicated bacterial extracts (25 µg equivalent protein content) were added to THP-1 growth medium. PBS treated cells were used as controls and GAPDH was used as a normalization control. Expression of A3F and A3G was measured using qRT-PCR. As shown in Fig. 1A, the expression of A3F and A3G mRNA was enhanced in the presence of the *S. cristatus* CC5A sonicated cell extract. Expression of A3F was increased as high as around 8 fold above baseline (set = 1), and expression of A3G was increased around 6 fold. In contrast, the extracts from the other oral bacteria tested and *E coli* DH5α (DH5A) had little or no effect on the expression of A3F and A3G. This experiment and all other experiments throughout this study were independently repeated at least three times. The variation was expressed by calculating the standard deviation (SD) from the three independent experiments and presented as the mean values ± SD. For the results of Western-blot analysis, one image representing the consent of the data will be shown.

We also tested several other oral *S. cristatus* strains such as CR311, CR3, CH34110 and pSH11a and pSH11b. CR3, CR311 and CH34110, which are closely related to CC5A, were as efficient as CC5A at inducing A3F and A3G expression, whereas pSH11b and pSH11a had almost no effect on APOBEC induction (Fig. 1B). Thus CC5A is not the only *S. cristatus* strain that is active, in terms of containing a molecule that induces APOBEC expression. We also treated THP-1 cells with different doses of CC5A in order to identify whether the induction of mRNA and protein expression levels was dose dependent. After a 16 h treatment, total RNA was isolated and induction of A3G and A3F measured by qRT-PCR. The induction of A3F and A3G mRNA gradually increased in response to the increasing amount of CC5A/PBS (Fig. 1C). By Western blot analysis, we also showed that A3G and A3F protein levels increased when THP-1 cells were treated with increasing amounts of CC5A/PBS (Fig. 1D). The dose response of APOBEC mRNA and protein further confirmed the effect of CC5A on A3F and A3G induction.

### CC5A/PBS contains a small active molecule, which covalently conjugates to the cell wall

**Figure 2:**
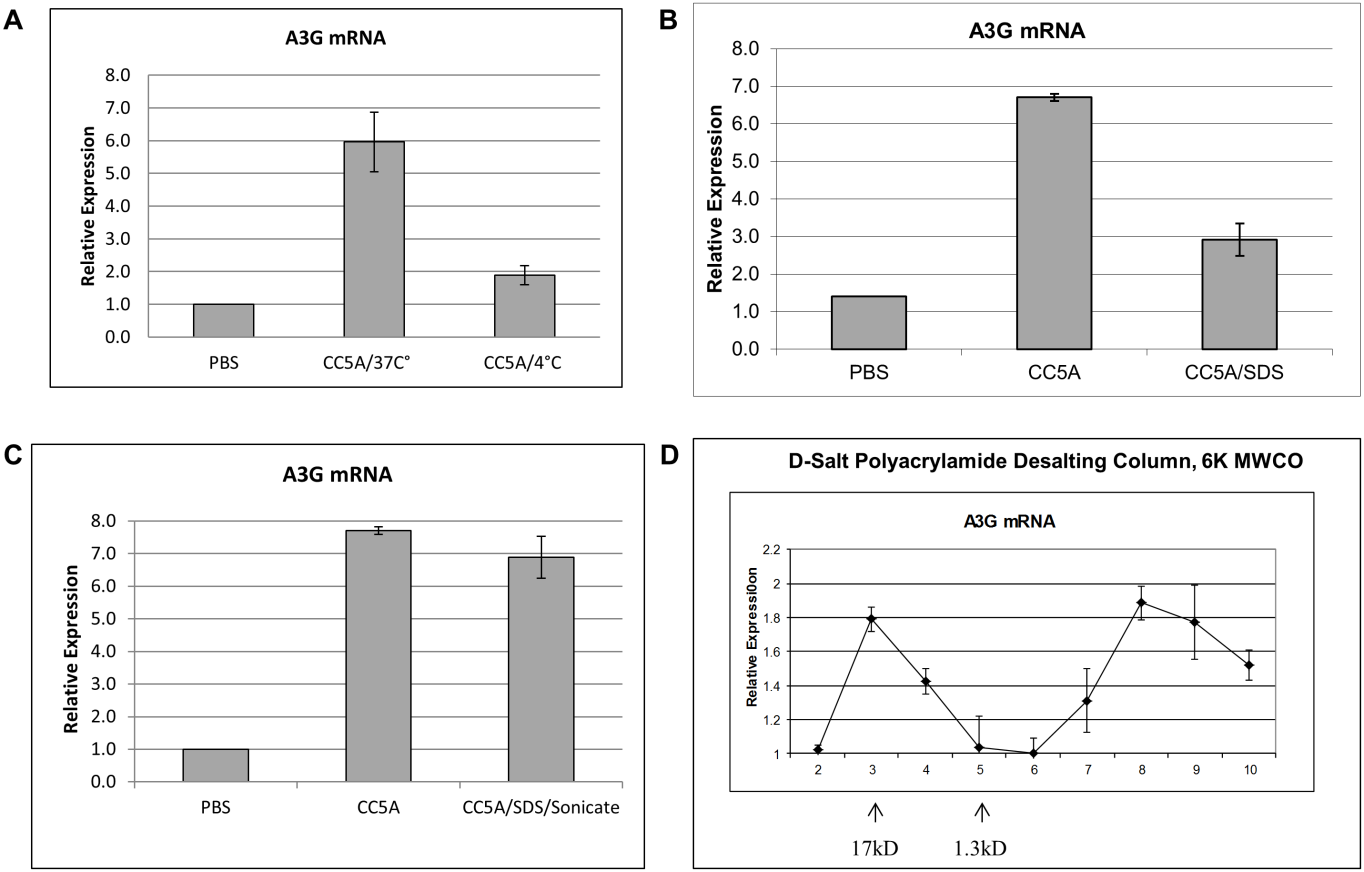
CC5A/PBS contains a small active heat-stable molecule, which may be covalently conjugated to the cell wall. (A) CC5A cells were incubated in PBS at 37°C or 4°C respectively. After 4 h of incubation, CC5A/PBS was prepared as described in Material and Methods. The CC5A/37°C and CC5A/4°C were used to treat THP-1 and the relative expression of A3G mRNA was measured by qRT-PCR. (B) CC5A cells were boiled in 2% SDS for 1 h. After being extensively washed with PBS, the SDS-treated CC5A cells were incubated in PBS at 37°C for 4 h for the preparation of CC5A/SDS. CC5A/SDS was used to treat THP-1 cells. The relative expression of A3G mRNA was measured by qRT-PCR. (C) After CC5A cells were treated with 2% SDS, the cells were sonicated to collect the CC5A extract as described in Material and Methods. The sonicated CC5A molecules were used to treat THP-1 cells. The relative expression of A3G mRNA was measured by qRT-PCR. (D) CC5A/PBS (100 µg) was subjected to a D-Salt Polyacrylamide Desalting Column (with a molecular weight cut-off of 6 kD). Two standards are shown by arrows: myoglobin (17 kD) and vitamin b12 (1.3 kD); 1.5 ml fractions were collected. The A3G induction response to eluate samples was measured by qRT-PCR. In all the qRT-PCR assays, the expression of A3G mRNA from untreated THP-1 cells was set as one.

Interestingly, we found that the active molecule appeared to be released from S. cristatus CC5A when the bacterial cells were simply incubated in PBS for 4 h at 37°C without sonication (Fig. 2A). When the process of 4 h incubation in PBS was done at 4°C (Fig. 2A) or after CC5A cells were boiled in 2% SDS for 1 h (Fig. 2B), the activity of inducing A3G expression was dramatically compromised. However, if sonication was used to treat the SDS treated CC5A cells, the active molecule was released into the supernatant (Fig. 2C). This indicates that the active molecule could not be removed from the cell (or destroyed) by boiling in 2% SDS, yet sonicating these cells released the active molecule into the PBS. This data implies that the active molecule may be covalently conjugated to the cell wall and an enzymatic reaction is necessary to release the active molecule from the CC5A cells when CC5A cells are simply incubated, for example, in PBS at 37°C for 4 h. These findings also show that the active molecule is very heat-stable.

To help identify the molecular weight of the active molecule, we used D-Salt Polyacrylamide Desalting Columns from Pierce with molecular weight cut-off of 6 kD. We first calibrated the column using Bio-Rad gel filtration standards, which contain equine myoglobin (17 kD) and vitamin B12 (1.3 kD). Equine myoglobin appears yellow and vitamin B12 appears pink in eluates. These two molecules eluted in fractions 3 and 5, respectively (Fig. 2D, indicated by arrows). When 100 µg CC5A/PBS was analyzed using this column, the peak A3G induction activity appeared in fractions 3 (17 kD) and 8 (Fig. 2D). A similar result was also observed for A3F induction (data not shown). Despite the fact that the molecular weight of fraction 8 must be <1.3 kD, this fraction is capable of inducing A3G mRNA. As the molecular weight cut-off of this column is 6 kD, the active molecule in fraction 3 is bigger than 6 kD and most likely represents the partially enzymatically digested cell wall component which is covalently conjugated to the small molecule found in fractions 8 and 9. This suggestion will be further addressed during our process of identifying this molecule. Nonetheless, the data shows that a small, heat stable molecule from CC5A is able to induce A3F and A3G expression.

### CC5A/PBS induces APOBEC through an MEK1/2 pathway

**Figure 3:**
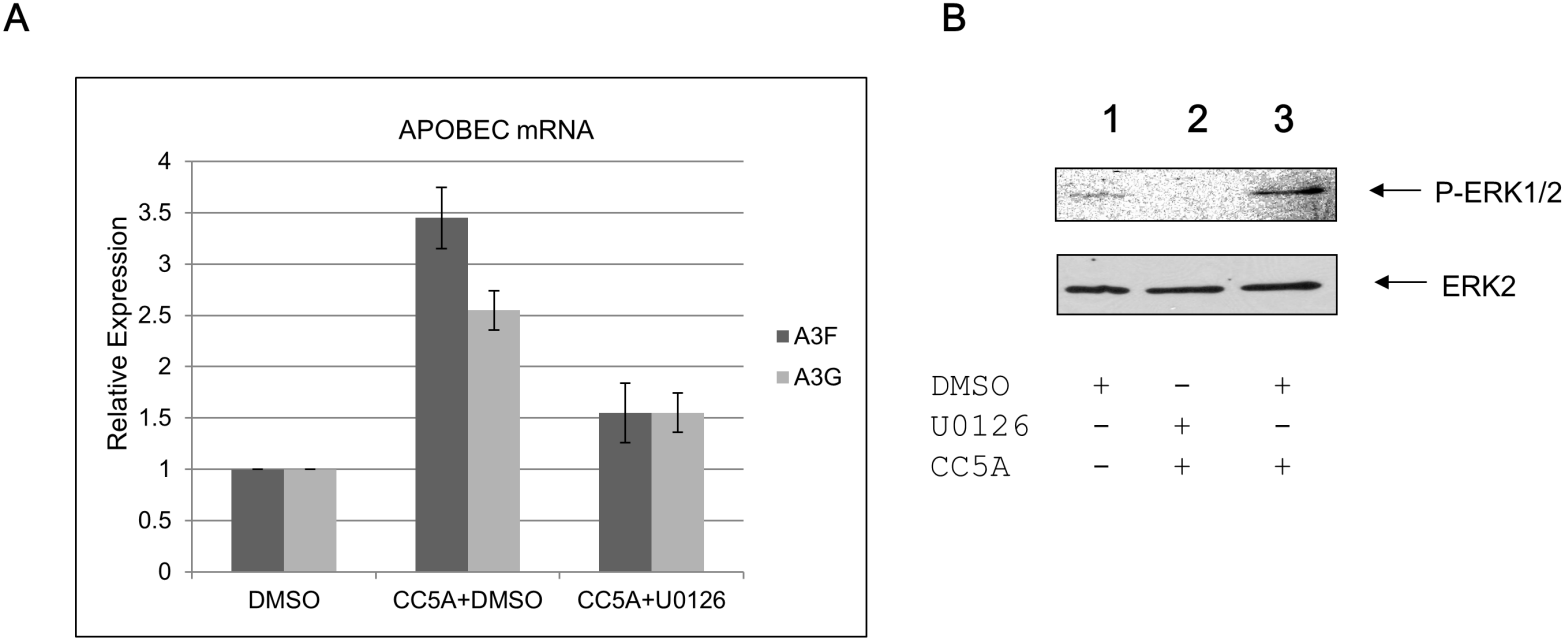
A3F and A3G are regulated through the MEK1/2 dependent pathway. (**A**) Prior to treatment with CC5A/PBS for 16 h, THP-1 cells were pre-treated by DMSO alone or the ERK1/2 inhibitor U0126 in DMSO for 1 h. The induction of A3G and A3F was measured by qRT-PCR. (B) Lysates from THP-1 cells pre-treated DMSO or U0126 in DMSO, following by CC5A/PBS (40 µg) are subjected to Western blot analysis. Phosphorylated ERK1/2 and ERK1/2 were probed by anti-p-ERK1/2 and anti-ERK2, respectively.

It has been reported that A3G is regulated through PKC and the JAK/MAPK signaling pathways in response to stimulation by PMA and cytokines [36], [37]. Stimulation of cell surface CCR5 and CD40 molecules by their ligands or by HSP70 up-regulates APOBEC3G expression in CD4^+^ T cells and dendritic cells through the p38 and ERK dependent signaling pathway [47]. A3G is also regulated through a STAT1-independent pathway when liver cells are treated with IFNα [39]. To test the signaling events involved in CC5A/PBS regulation of APOBEC expression, THP-1 cells were pretreated with the MEK inhibitor U0126. U0126 strongly inhibited CC5A/PBS mediated up-regulation of A3F and A3G mRNA (Fig. 3A). To confirm that CC5A/PBS activates the MAPK pathway, we analyzed the expression of phospho-ERK1/2 by using Western blot analysis. After THP-1 cells were treated with CC5A/PBS, phospho-ERK1/2 (Fig. 3B lane 3) was significantly increased compared with PBS treated THP-1 cells (lane 1). When U0126 was used to pretreat THP-1 cells, phospho-ERK1/2 was undetectable (lane 2) even with CC5A/PBS treatment. We noticed that the phosphorylation of ERK1/2 in lane 2 was even lower than that in lane 1 in Fig. 3B, indicating that U0126 not only inhibited the phosphorylation of ERK1/2 induced by CC5A, but also the phosphorylation caused by the endogenous mechanisms. Taken together, these results indicate that CC5A/PBS regulates A3G and A3F through a MEK1/2 dependent pathway.

### CC5A/PBS reduces HIV-1 infectivity and inhibits HIV-1 replication

**Figure 4:**
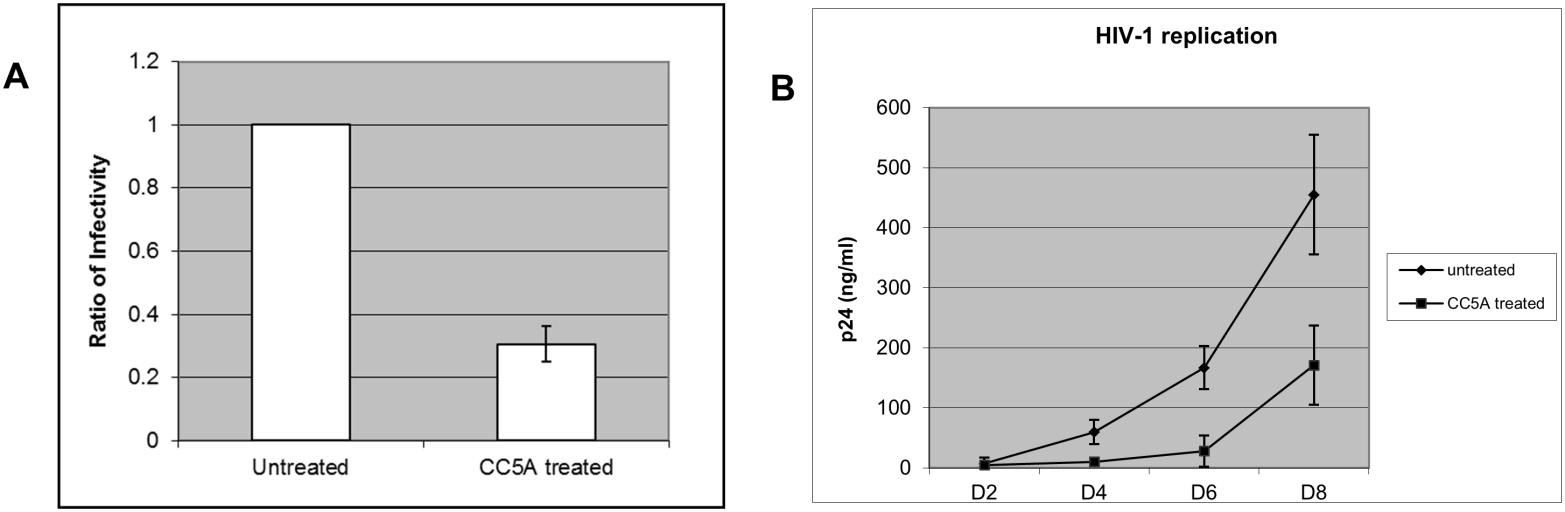
The active CC5A molecule inhibits HIV-1 replication. (A) THP-1/IIIB cells were treated with PBS or CC5A/PBS for 16 h. Post-treatment, THP-1 cells were washed twice by PBS and cultured for 24 h. The cells were then washed with PBS and cultured for another 24 h. The culture supernatants were collected for infectivity assays. The amount of input virus for the infectivity assay was normalized by p24 ELISA. (B) THP-1 cells were pre-treated overnight with CC5A/PBS or PBS before IIIB infection. After 3 h of infection, the cells were washed twice with PBS and put back into culture for eight days. Viral samples were collected every other day and viral replication was monitored by p24 assay.

A3G is a potent host restriction factor of HIV-1 replication. It reduces HIV-1 infectivity once it is packaged into HIV-1 particles [15]. Since CC5A/PBS induces A3G expression, we next asked whether CC5A/PBS can reduce HIV infectivity in the THP-1 cell system. THP-1/IIIB cells, chronically infected by HIV-1 IIIB, were treated with PBS or CC5A/PBS (40 µg) for 16 h. Post-treatment, THP-1 cells were washed twice by PBS and cultured for 24 h. After 24 h, the cells were washed with PBS again and cultured for another 24 h. The culture supernatant was collected for a MAGI infectivity assay as describe previously [48]. The amount of virus for the infectivity assay was normalized by p24 ELISA [48]. After CC5A/PBS treatment, HIV infectivity was reduced approximately 70% compare to untreated cells (Fig. 4A). To determine if CC5A/PBS was capable of inhibiting HIV-1 replication throughout multiple rounds of infection, THP-1 cells were pretreated overnight with CC5A/PBS (40 µg) or PBS before infection with HIV-1 IIIB. After 3 h of infection, the cells were washed twice with PBS and put back into culture for eight days. Viral-containing supernatants were collected every two days and viral replication was monitored by p24 ELISA. Viral replication in CC5A/PBS-treated THP-1 cells was significantly inhibited (Fig. 4B). This data suggests that CC5A/PBS is able to inhibit HIV-1 replication.

### A3G knockdown compromised the effect of CC5A in reducing HIV infectivity

**Figure 5:**
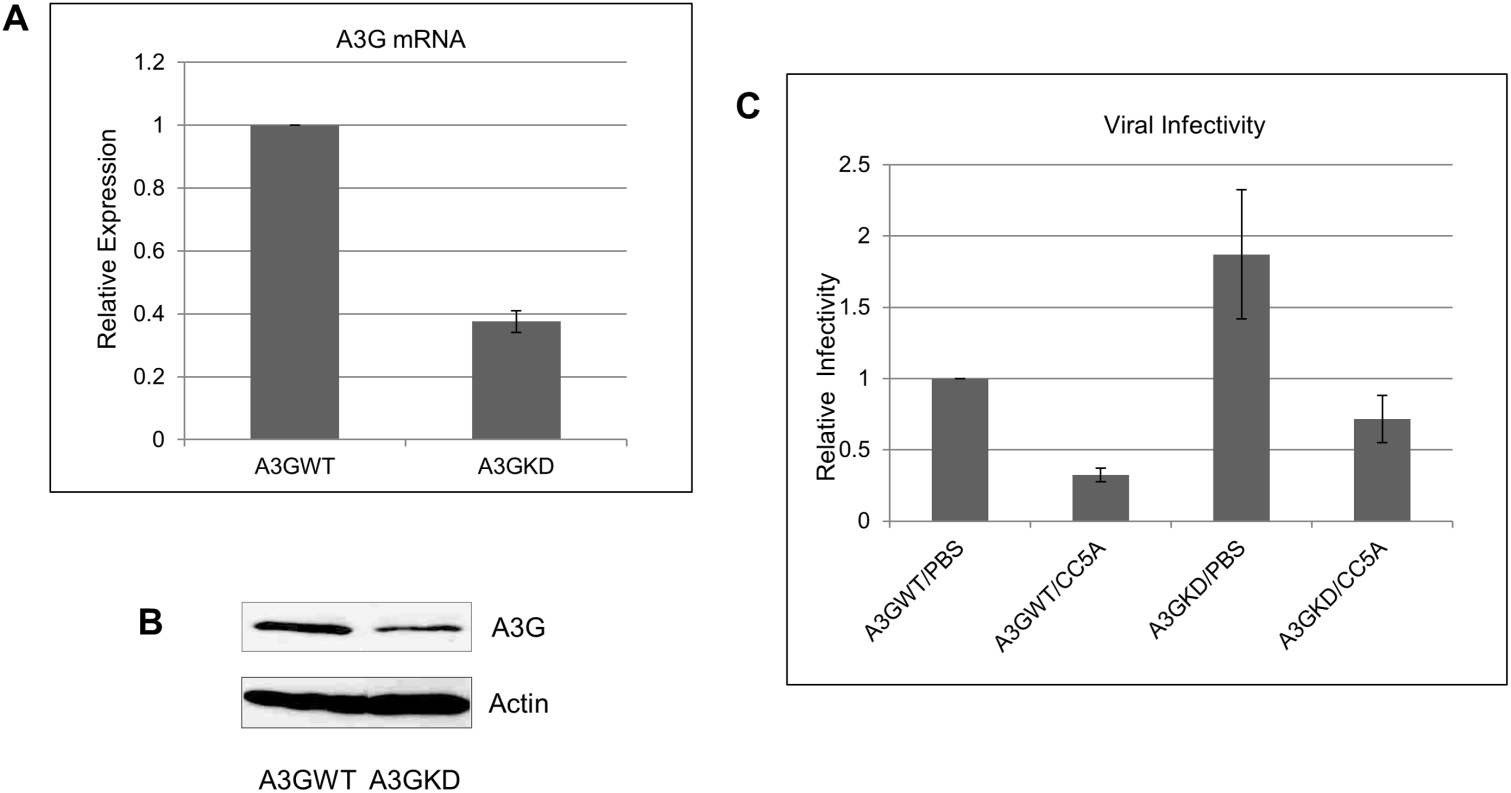
A3G knockdown compromised the effect of CC5A in reducing HIV infectivity. pX330-A3G1 and pX330-A3G2 (3 µg each) were transfected into 1×10^6^ THP-1 cells using Neon Transfection System (Invitrogen). Three days post transfection, the transfected THP-1 cells were cloned by limited dilution method. Single clones were screen by Western-blot and qRT-PCR for A3G expression analysis. A3GKD was selected as A3G knockdown cell line for further experiment. A3G expression was tested by Western-blot (A) and qRT-PCR (B) analysis. THP-1/IIIB and THP-1 A3GKD/IIIB were treated with CC5A or PBS to measure the effects of CC5A on reducing HIV infectivity in THP-1 and THP-1 A3GKD cell line (B).

CC5A induced A3G expression (Fig. 1) and inhibits HIV infectivity (Fig. 4). This suggests that A3G may play a role in mediating the effect of CC5A in inhibiting HIV infectivity. To prove this, we established an A3G knockdown cell line using CRISP/Case 9 system. CRISPR stands for Clustered Regularly Interspaced Short Palindromic Repeats. The CRISPR/Case system is currently the most commonly used RNA-Guided Endonuclease technology for genome engineering [49], [50]. We constructed plasmids pX300-A3G1 and pX300-A3G2, which targeted A3G genome exon 3. We established A3G knockdown cell line after one round screening as described in the material and methods section. As shown in Fig. 5A and 5B, the expression level of A3G mRNA and protein levels decreased more than 50% in THP-1 A3GKD cell line compared with wild-type THP-1. Both THP-1 and THP-1 A3GKD cell lines were infected with HIV-1 IIIB to generate chronically infected cell lines. The effects of CC5A on reducing HIV infectivity were measured in both cell lines as described earlier for Fig. 4A. As shown in Fig. 5C, the viral infectivity of PBS treated THP-1/A3GKD/PBS is about two fold higher than the infectivity of PBS treated wild-type THP-1. This data is consistent with the function of A3G in which lower expression of A3G will render higher viral infectivity. When the infectivity of CC5A treated THP-1 A3GKD was compared with wild-type THP-1, we demonstrate that the infectivity of THP-1 A3GKD was about two fold higher than the infectivity of CC5A treated wild-type THP-1. The data showed that A3G knockdown reduced CC5A’s effects in reducing HIV infectivity, suggesting that A3G played a role in mediating the effects of CC5A in inhibiting HIV infectivity.

### CC5A enhances HIV G to A hypermutation rate

**Figure 6:**
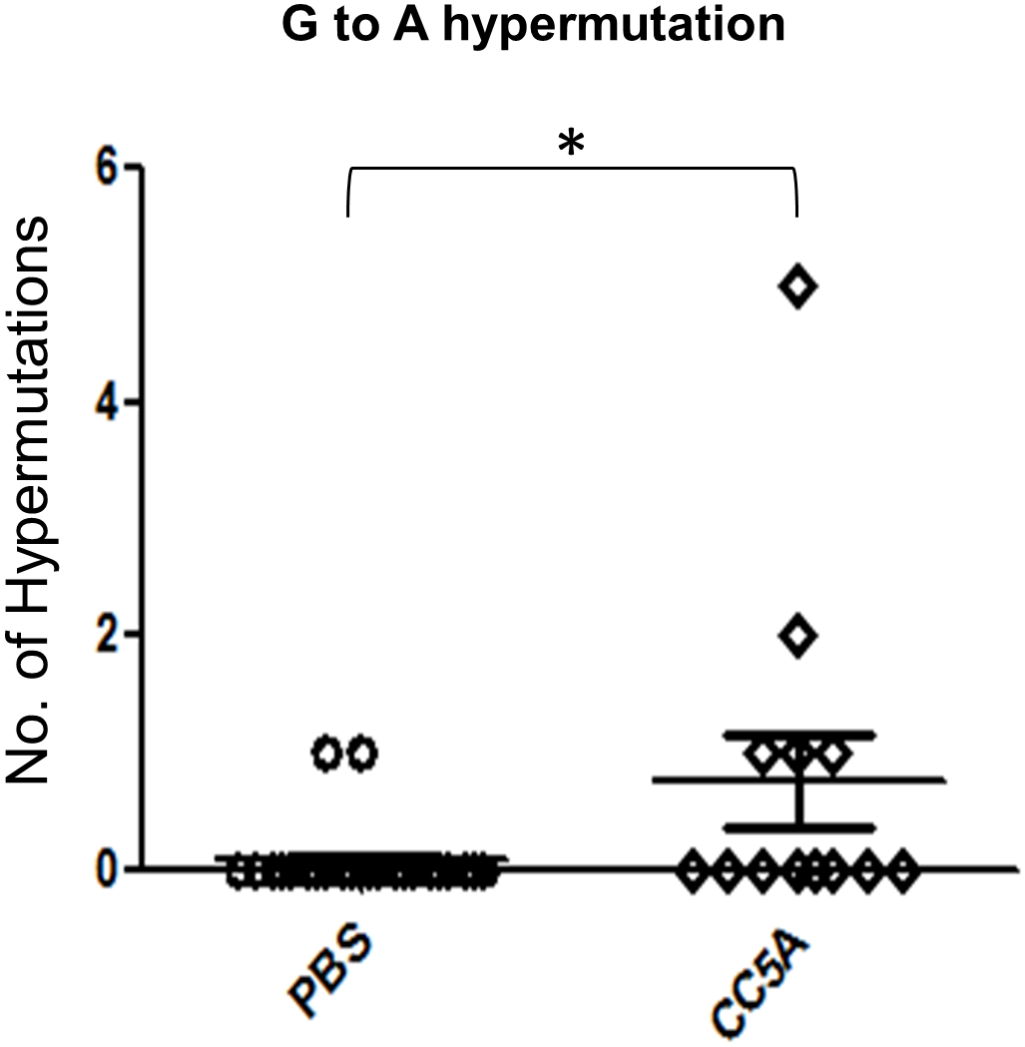
CC5A enhances HIV G to A hypermutation rate. HIV from CC5A or PBS treated THP-1 culture supernatants were used to infect SupT1 cells. The infected SupT1 cells were washed twice with PBS then incubated at 37°C, 5% CO2 for 16 h. HIV cDNA was isolated using DNeasy DNA isolation kit (Qiagen). A 650 bp DNA fragment covering a portion of nef, U3 and R of HIV-1 was amplified by PCR. The PCR products were cloned into the TOPO TA-cloning vector. The nucleotide sequences of individual clones from each infected culture sample were determined. Statistical significance was determined by Mann Whitney U test using GraphPad Prism software. Mann Whitney U test was used to calculate p-value.

A G to A hypermutation is a hallmark of APOBEC antiviral activity. We therefore performed a G to A hypermutation analysis to determine if this function of APOBEC contributes to CC5A mediated inhibition of HIV replication. As mentioned in the material and methods, culture supernatants were used to infect SupT1 cells for a DNA hypermutation assay. Only 2 G to A hypermutation was detected in 24 clones from SupT1 cells infected with virus taken from the untreated THP-1/IIIB cell line. Infection of the SupT1 cells with virus obtained from the CC5A/PBS-treated THP-1/IIIB cell line resulted in 9 hypermutations in 13 clones upon examination of viral DNA (Fig. 6). Mann Whitney U test calculated a p-value of 0.023. Thus CC5A/PBS significantly increased G to A hypermutations in HIV-1 IIIB viral DNA. The data suggest that APOBEC plays a role in CC5A/PBS mediated-inhibition of HIV-1 replication.

### CC5A/PBS induces A3G and A3F expression in human oral OKF6 keratinocytes and human vaginal VK2 epithelial cells

**Figure 7:**
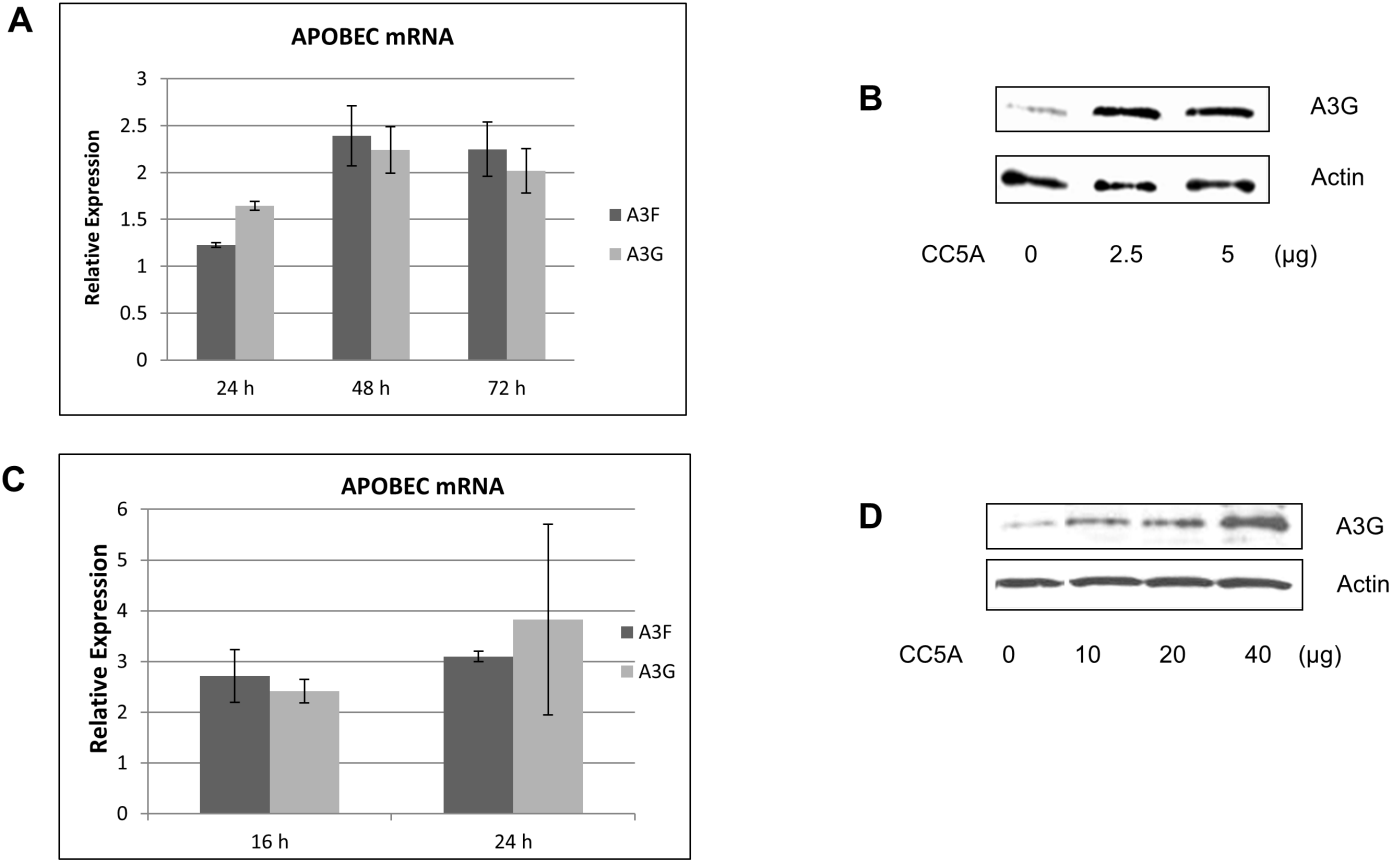
CC5A/PBS induces A3G and A3F expression in human oral keratinocyte (OKF6) and human vaginal VK2 epithelial cells. (**A**) CC5A/PBS (5 µg) was used to treat OKF6 cells. The cells were harvested at the indicated time points, and qRT-PCR was used to measure the relative expression of A3F and A3G mRNA. The expression levels shown represent the ratio of CC5A/PBS treated cells compared to the untreated cells at each given time point. (**B**) Different doses (0–5 µg) of CC5A/PBS were used to treat OKF6 cells for 72 h, and A3G expression was measured by Western-blot analysis. (**C**) VK2 cells were treated with 40 µg CC5A/PBS or vehicle for the time periods shown. qRT-PCR was used to measure the relative expression of A3F and A3G mRNA in these cells. The expression levels shown represent the ratio of the levels found in CC5A/PBS treated cells compared to untreated cells at each time point. (**D**) Different doses (0–40 µg) of CC5A/PBS were used to treat VK2 cells for 48 h. A3G protein expression was measured by Western-blot analysis.

Vaginal epithelial cells and oral keratinocytes are the first line of defense after vaginal or oral exposure of HIV-1 to females. It has been shown that mouse APOBEC3 plays a role in blocking the transmission of mouse mammary tumor virus [51] and murine acquired immunodeficiency virus [52]. Therefore, we determined if CC5A/PBS also induced A3G and A3F expression in VK2 and OKF6 cells. In a pilot study, we found OKF6 cells were very sensitive to CC5A treatment. We therefore used a low dose of CC5A/PBS (5 µg) to treat OKF6 cells. Cells were harvested at the indicated time points (Fig. 7A) and the relative APOBEC mRNA levels measured by qRT-PCR. CC5A/PBS increased the APOBEC A3F and A3G mRNA levels up to 2.5 fold at 48 h post treatment compare to untreated cells. The values at 72 h were similar to those at 48 h. Untreated samples in Fig. 6A were normalized to 1.0. The effect of 2.5 and 5 µg of CC5A/PBS on A3G protein levels at 72 h is shown in Fig. 7B. Therefore as little as 2.5 µg was as effective as 5 µg in these cells. Similarly, CC5A/PBS (40 µg) also increased A3F and A3G mRNA (Fig. 7C) in VK2 cells compared to controls (set at a comparative value of 1.0). The effect of CC5A appears to some degree, cell specific, as the sensitivity to CC5A/PBS with respect to induction of A3G protein levels was greatest in THP-1>VK2>OKF6 cells (Fig. 7). In addition, 40 µg CC5A incubated for 16 h with THP-1 cells produced an 8 fold increase in A3G mRNA, the same dose of CC5A produced a<2.5 fold difference at 16 h in VK2 cells.

### CC5A/PBS and Sonicated CC5A do not cause cytotoxicity in THP-1 cells

**Figure 8:**
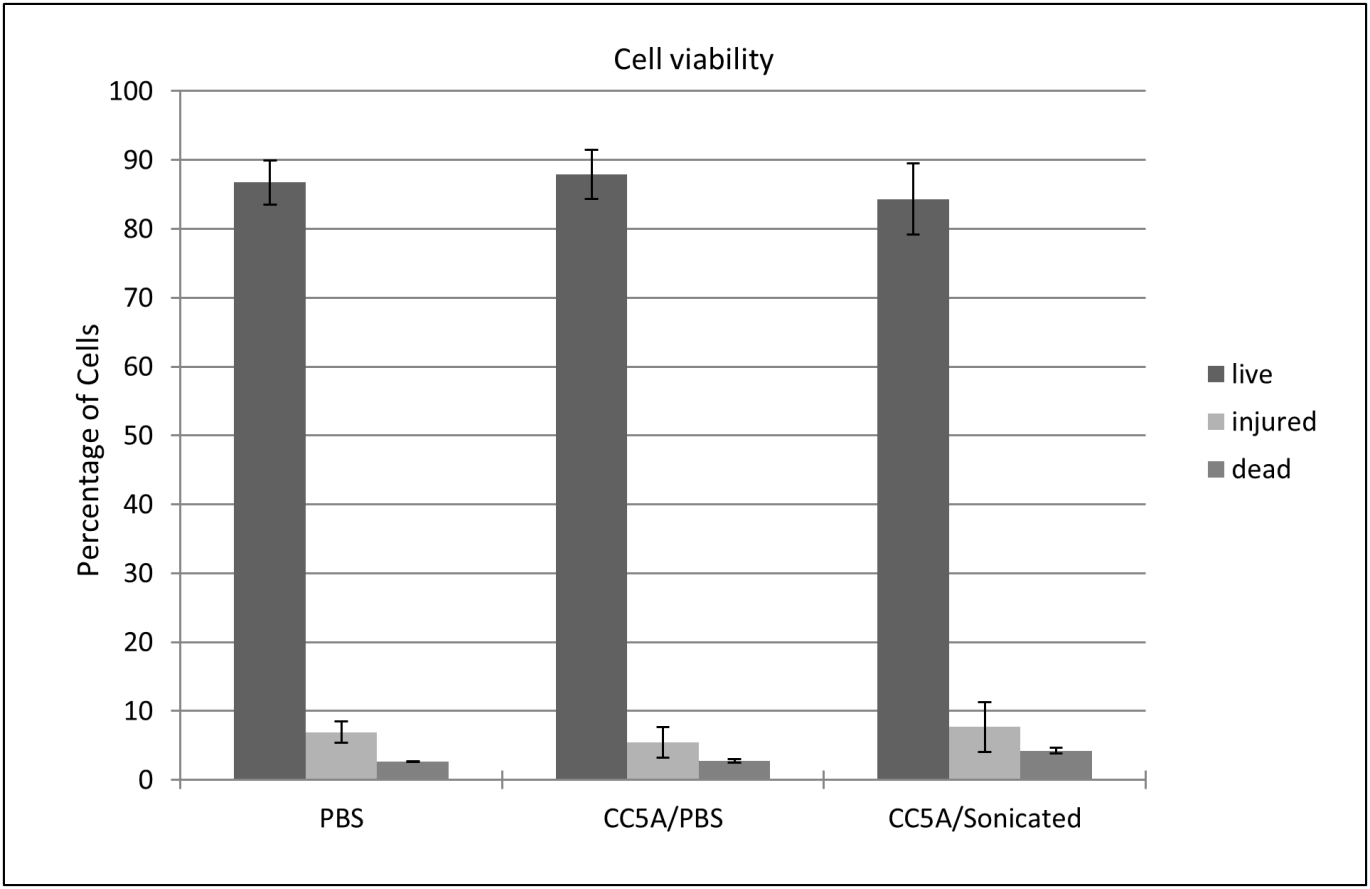
CC5A/PBS and sonicated CC5A do not cause cytotoxicity in THP-1 cells. The cytotoxicity of CC5A/PBS and sonicated CC5A treatment was measured by a Live/Dead Cell Vitality Assay Kit (Invitrogen). THP-1 cells were treated with PBS, 40 µg CC5A/PBS and 25 µg sonicated CC5A for 16 h then subjected to the Live/Dead Cell Vitality Assay Kit analysis.

The cytotoxicity of CC5A/PBS and Sonicated CC5A treatment was measured by a Live/Dead Cell Vitality Assay Kit (Invitrogen). The kit provides a two-color fluorescence assay that distinguishes metabolically active cells from injured cells and dead cells. THP-1 cells were treated with PBS (untreated control), 25 µg sonicated CC5A or 40 µg CC5A/PBS respectively (Fig. 8). Cells were harvested 16 h post-treatment. The cytotoxicity effect of CC5A treatment was measured by a Live/Dead Cell Vitality Assay kit following the manufacturer’s directions. While untreated controls indicated the presence of around 85% live cells, when treated with sonicated CC5A or CC5A/PBS, the percent of live THP-1 cells was still 85% (Fig. 8). In addition, percentage of the injured and dead cells are all similar among the three samples (Fig. 8). In summary, the treatment of sonicated CC5A or CC5A/PBS does not cause appreciably cytotoxicity to THP-1 cells.

## Discussion

Although most HIV-1 is transmitted through mucosal surfaces, the transmission through the oral mucosa is uncommon. Further HIV from the saliva of an HIV-seropositive individual is less infectious than that from plasma or vaginal fluid. The recovery rate of infectious HIV from saliva is low (1–2%) in HIV-seropositive individuals, although the DNA and RNA detection frequency of HIV by PCR is higher (20–50%) [53], suggesting there are innate inhibitory factors in saliva and/or in the oral epithelium that function to reduce HIV infectivity. Numerous studies have shown that oral transmission is not an independent risk factor for an HIV-seronegative individual (see review in [54]). In the oral cavity, HIV host restriction factors, such as secretory leukocyte protease inhibitor (SLPI), lysozyme, defensins, ribonuclease and thrombospondin-1, may be contributors to the extremely low risk of oral transmission of the virus [54]. In this study, we show that a molecule from *S. cristatus* up-regulates expression levels of A3G and A3F. This molecule also inhibits HIV-1 replication in an APOBEC-related manner (Figs. 4, 5, 6). Indeed, the fold-reduction of HIV infectivity in CC5A -treated A3GKD cells relative to PBS-treated A3GKD cells is approximately the same as that in CC5A-treated A3GWT cells relative to PBS-treated A3GWT cells (Fig. 5C). However, considering the fact that the infectivity of CC5A treated THP-1 A3GKD was about two fold higher than the infectivity of CC5A treated wild-type THP-1, we think the A3G knockdown data demonstrated that A3G played a role in mediating the effects of CC5A in inhibiting HIV infectivity. Similar data was obtained when Peng et al. tried to prove that A3G played a role in IFN induced ant-HIV-1 activity [55]. These findings support the idea that APOBEC3 proteins may represent an additional set of host restriction factors that work to block the oral transmission of HIV. Indeed, it has been shown that mouse APOBEC3 plays a role in blocking the transmission of mouse mammary tumor virus (46) and murine acquired immunodeficiency virus (47). In addition, our data demonstrate that the CC5A-derived molecule is able to induce A3G and A3F expression in oral keratinocytes (OKF6) and vaginal epithelial cells (VK2). Studies in relevant primary cells and using an HIV-1 transmission assay will bolster the clinical relevance of our results to date.

Previous reports show that A3G expression is regulated by PMA, PHA and certain cytokines through the PKC/JAK/ERK/MAPK signaling pathway [24], [36], [37]. Stimulation of cell surface CCR5 and CD40 molecules by their ligands or by HSP70 up-regulates APOBEC3G expression in CD4^+^ T cells and dendritic cells through the p38 and ERK dependent signaling pathway [47]. In our study, we show that a molecule from *S. cristatus* up-regulates A3G and A3F through the same pathway. To our knowledge, this is the first report showing that A3G and A3F expression may be regulated by an oral bacterial component. In response to bacterial exposure, host immune systems rapidly induce the expression of a variety of genes involved in producing interferons and proinflammatory cytokines to generate protection against bacterial invasion. The reason why A3G and A3F are also up-regulated and whether they play a role in the protection against bacterial invasion are still open questions.

This active molecule could be a potential candidate for developing novel anti-HIV drugs to prevent or treat HIV infection. *S. cristatus* CC5A is a gram positive bacterium. It exists in normal, healthy oral cavities. It has not been associated with any oral infectious disease. Therefore, it is highly possible that this active molecule could be useful because of its low toxicity. In fact, our cytotoxicity data showed there was no obvious cytotoxicity after THP-1 cells were treated for 16 h with preparations containing the *S. cristatus* CC5A active molecule. Boiling in 2% SDS was not able to inactive the molecule suggests that the active molecule is heat-stable. In addition given the fact that the active molecule is smaller than 1.3 kD, we argue that *S. cristatus* CC5A contains a small, heat-stable molecule, which has the effect to enhance innate immunity and inhibit HIV-1 replication. The idea of promoting A3G and A3F as anti-HIV/AIDS agents is distinct from conventional anti-HIV drugs, such as reverse transcriptase and protease inhibitors, wherein HIV is prone to generating drug-resistant mutations. A3G interacts with the HIV nucleocapsid and is incorporated into the virion [56]–[63]. The domain on nucleocapsid with which A3G interacts is critical for HIV assembly, implying the impossibility of HIV to exclude A3G by mutation. On the other hand, it has been suggested that HIV actively recruits a small amount of A3G to promote its diversification, escape immune pressure and generate drug resistance [64]–[66]. This HIV reliance on low-levels of APOBEC can be exploited by designing an anti-HIV/AIDS drug based on this CC5A extract that amplifies A3G and A3F expression beyond what the virus can sustain. Continuation of these studies may result in an entirely new class of antiretroviral drugs.
